# Supplementary material for: Field Trial Vaccination against Cowpox in Two Alpaca Herds
Source: Viruses. 2020 Feb 20;12(2):234. doi: 10.3390/v12020234 (PMC7077317; doi:10.3390/v12020234)
Supplement: Supplementary file 1 [file viruses-12-00234-s001.pdf]

Table S1: Individual antibody titres over time after using a prime and boost MVA vaccination regimen against cowpox in two alpaca herds in Germany

ID	Herd	Gr	P	Age in years	Sex	Pre-existing field titre		IFA (MVA)			
						IFA (CPXV)*	IFA (MVA)**	TP2	TP3	TP4	TP5
**101**	I	1	1	4.25	f	1:500	1:500	1:2,000	1:500	1:200	1:200
**103**	I	1	1	7.45	f	1:500	1:2,000	1:4,000	1:4,000	1:1,000	1:1,000
**118**	I	1	1	0.15	m		n.d.	1:200	1:4,000	1:200	1:200
**136**	I	1	1	5.33	f		1:4000	1:4,000	1:8,000	1:2,000	1:2,000
**138**	I	1	1	6.57	f	1:500	1:4000	1:1,000	1:2,000	1:500	1:500
**139**	I	1	1	10.49	f	1:500	>0<1:200	1:500	1:500	1:200	1:200
**145**	I	1	1	8.50	f	1:200	1:500	1:4,000	1:2,000	1:500	1:1,000
**166**	I	1	1	1.53	m	1:500	1:2,000	1:4,000	1:8,000	1:2,000	1:1,000
**1**	II	1	1	2.99	f		<1:200	1:1,000	1:500	1:500	1:500
**2**	II	1	1	5.01	f		<1:200	1:1,000	1:1,000	1:1,000	1:200
**3**	II	1	1	3.79	f		<1:200	1:1,000	1:500	1:500	1:200
**12**	II	1	1	1.84	f	1:500	1:4,000	1:1,000	1:1,000	1:500	1:500
**15**	II	1	1	3.98	f		<1:200	1:8,000	1:4,000	1:2,000	1:1,000
**16**	II	1	1	1.86	f	1:500	1:8,000	1:4,000	1:2,000	1:2,000	1:1,000
**17**	II	1	1	6.31	f		<1:200	1:4,000	1:2,000	1:1,000	1:500
**20**	II	1	1	4.00	f		<1:200	1:2,000	1:1,000	1:200	1:500
**25**	II	1	1	2.75	f		<1:200	1:1,000	1:500	1:200	1:200
**112**	I	2	2	0.11	m		n.d.	1:500	1:500	1:500	>0<1:200
**9**	II	2	2	2.08	m		<1:200	1:200	1:4,000	1:200	>0<1:200
**8**	II	2	3	2.00	m		<1:200	<1:200	1:4,000	1:500	1:500
**165**	I	2	4	1.75	m	1:500	<1:200	1:1000	1:1,000	<1:200	<1:200
**105**	I	2	4	4.34	f		<1:200	1:200	1:500	<1:200	<1:200
**109**	I	2	4	0.19	f		n.d.	1:200	1:2,000	<1:200	<1:200
**123**	I	2	4	4.25	f		<1:200	1:200	1:200	<1:200	<1:200
**125**	I	2	4	1.74	f		<1:200	1:200	1:1,000	<1:200	<1:200
**131**	I	2	4	0.31	f		n.d.	1:200	1:2,000	<1:200	<1:200
**133**	I	2	4	1.24	f		<1:200	1:200	1:500	<1:200	<1:200
**135**	I	2	4	5.62	f		<1:200	1:500	1:200	<1:200	<1:200
**150**	I	2	4	1.28	f		<1:200	1:200	1:500	<1:200	<1:200
**154**	I	2	4	4.23	m		<1:200	1:200	1:500	<1:200	<1:200
**158**	I	2	4	7.26	m		<1:200	1:200	1:500	<1:200	<1:200
**159**	I	2	4	2.29	m		<1:200	1:200	1:500	<1:200	<1:200
**162**	I	2	4	1.56	m		<1:200	1:200	1:200	<1:200	<1:200
**120**	I	2	5	0.31	f		n.d.	<1:200	1:500	1:200	<1:200
**13**	II	2	6	0.91	m		<1:200	>0<1:200	1:1,000	>0<1:200	<1:200
**21**	II	2	6	6.01	f		<1:200	>0<1:200	1:1,000	>0<1:200	<1:200
**116**	I	2	7	0.13	m		n.d.	<1:200	1:500	>0<1:200	<1:200
**27**	II	2	7	4.84	f		<1:200	<1:200	1:1,000	>0<1:200	<1:200
**128**	I	2	8	1.69	f		<1:200	>0<1:200	1:500	<1:200	<1:200
**134**	I	2	8	2.52	f		<1:200	>0<1:200	1:1000	<1:200	<1:200
**147**	I	2	8	0.32	m		n.d.	>0<1:200	1:200	<1:200	<1:200
**161**	I	2	8	1.68	m		<1:200	>0<1:200	1:500	<1:200	<1:200
**18**	II	2	8	0.88	f		<1:200	>0<1:200	1:200	<1:200	<1:200
**102**	I	2	9	0.12	m		n.d.	<1:200	1:200	<1:200	<1:200
**106**	I	2	9	4.42	f		<1:200	<1:200	1:200	<1:200	<1:200
**107**	I	2	9	0.07	f		n.d.	<1:200	1:500	<1:200	<1:200
**111**	I	2	9	4.00	f		<1:200	<1:200	1:500	<1:200	<1:200

**113**	I	2	9	4.25	f		<1:200	<1:200	1:200	<1:200	<1:200
**114**	I	2	9	0.08	m		n.d.	<1:200	1:1,000	<1:200	<1:200
**117**	I	2	9	4.25	f		<1:200	<1:200	1:500	<1:200	<1:200
**122**	I	2	9	1.75	f		<1:200	<1:200	1:500	<1:200	<1:200
**124**	I	2	9	2.13	f		<1:200	<1:200	1:500	<1:200	<1:200
**129**	I	2	9	1.85	f		<1:200	<1:200	1:500	<1:200	<1:200
**130**	I	2	9	4.25	f		<1:200	<1:200	1:500	<1:200	<1:200
**132**	I	2	9	2.37	f		<1:200	<1:200	1:200	<1:200	<1:200
**142**	I	2	9	4.42	f		<1:200	<1:200	1:500	<1:200	<1:200
**146**	I	2	9	4.25	f		<1:200	<1:200	1:500	<1:200	<1:200
**148**	I	2	9	2.50	f		<1:200	<1:200	1:500	<1:200	<1:200
**156**	I	2	9	3.36	m		<1:200	<1:200	1:200	<1:200	<1:200
**157**	I	2	9	2.32	m		<1:200	<1:200	1:200	<1:200	<1:200
**160**	I	2	9	1.27	m		<1:200	<1:200	1:200	<1:200	<1:200
**164**	I	2	9	1.64	m		<1:200	<1:200	1:500	<1:200	<1:200
**167**	I	2	9	1.76	m		<1:200	<1:200	1:500	<1:200	<1:200
**7**	II	2	9	9.76	m		<1:200	<1:200	1:500	<1:200	<1:200
**14**	II	2	9	4.77	f		<1:200	<1:200	1:500	<1:200	<1:200
**19**	II	2	9	0.94	f		<1:200	<1:200	1:200	<1:200	<1:200
**22**	II	2	9	15.45	f		<1:200	<1:200	1:500	<1:200	<1:200
**108**	I	2	10	4.25	f		<1:200	1:200	<1:200	<1:200	<1:200
**149**	I	3	11	4.34	f		<1:200	>0<1:200	>0<1:200	<1:200	<1:200
**104**	I	3	12	0.07	f		n.d.	<1:200	>0<1:200	<1:200	<1:200
**115**	I	3	12	5.08	f		<1:200	<1:200	>0<1:200	<1:200	<1:200
**121**	I	3	12	3.35	f		<1:200	<1:200	>0<1:200	<1:200	<1:200
**140**	I	3	12	4.34	f		<1:200	<1:200	>0<1:200	<1:200	<1:200
**153**	I	3	12	7.37	m		<1:200	<1:200	>0<1:200	<1:200	<1:200
**24**	II	3	12	11.45	f		<1:200	<1:200	>0<1:200	<1:200	<1:200
**126**	I	3	13	3.22	f		<1:200	<1:200	<1:200	<1:200	<1:200
**127**	I	3	13	0.38	m		n.d.	<1:200	<1:200	<1:200	<1:200
**143**	I	3	13	0.34	f		n.d.	<1:200	<1:200	<1:200	<1:200
**151**	I	3	13	2.50	f		<1:200	<1:200	<1:200	<1:200	<1:200
**163**	I	3	13	1.64	m		<1:200	<1:200	<1:200	<1:200	<1:200
**23**	II	3	13	6.75	f		<1:200	<1:200	<1:200	<1:200	<1:200
**155**	I	4	14	4.97	m		<1:200	1:500	1:200	<1:200	1:200
**152**	I	4	15	1.26	f		<1:200	<1:200	1:500	<1:200	1:200
**10**	II	4	15	2.02	m		<1:200	<1:200	1:2,000	<1:200	1:200
**137**	I	4	16	1.09	f		n.d.	<1:200	1:200	<1:200	>0<1:200
**11**	II	4	16	10.03	m		<1:200	<1:200	1:500	<1:200	>0<1:200
**6**	II	4	17	8.55	m		<1:200	1:200	1:1,000	<1:200	>0<1:200
**144**	I	4	18	1.12	f		n.d.	>0<1:200	1:200	<1:200	>0<1:200

ID, animal identity number; Herd: I – Thuringia (n = 64); II – Saxony Anhalt (n = 24); Gr, group; P, individual titre changes; IFA, indirect immunofluorescence assay (endpoint dilution); <1:200, dilution 1:200 tested negative; >0<1:200, dilution 1:200 tested intermediate; CPXV, cowpox virus; MVA, modified vaccinia virus Ankara; TP, time point (2 – four weeks after initial vaccination, 3 – four weeks after booster vaccination, 4 – six months after booster vaccination, 5 – 12 months after boost vaccination); n.d., not done (animal had not been born or was too young for sampling at the time of previous investigations performed by [16]); * results according to [16]; ** serum samples collected in a previous study [16], re-tested using IFA (MVA)
